# Increasing the Content of Olive Mill Wastewater in Biogas Reactors for a Sustainable Recovery: Methane Productivity and Life Cycle Analyses of the Process

**DOI:** 10.3390/foods10051029

**Published:** 2021-05-10

**Authors:** Souraya Benalia, Giacomo Falcone, Teodora Stillitano, Anna Irene De Luca, Alfio Strano, Giovanni Gulisano, Giuseppe Zimbalatti, Bruno Bernardi

**Affiliations:** Dipartimento di Agraria, Università degli Studi Mediterranea di Reggio Calabria, Località Feo di Vito, 89122 Reggio Calabria, Italy; soraya.benalia@unirc.it (S.B.); giacomo.falcone@unirc.it (G.F.); anna.deluca@unirc.it (A.I.D.L.); astrano@unirc.it (A.S.); ggulisano@unirc.it (G.G.); gzimbalatti@unirc.it (G.Z.); bruno.bernardi@unirc.it (B.B.)

**Keywords:** anaerobic codigestion, biomethane, life cycle assessment (LCA), life cycle costing (LCC), olive mill by-products

## Abstract

Anaerobic codigestion of olive mill wastewater for renewable energy production constitutes a promising process to overcome management and environmental issues due to their conventional disposal. The present study aims at assessing biogas and biomethane production from olive mill wastewater by performing biochemical methane potential tests. Hence, mixtures containing 0% (blank), 20% and 30% olive mill wastewater, in volume, were experimented on under mesophilic conditions. In addition, life cycle assessment and life cycle costing were performed for sustainability analysis. Particularly, life cycle assessment allowed assessing the potential environmental impact resulting from the tested process, while life cycle costing in conjunction with specific economic indicators allowed performing the economic feasibility analysis. The research highlighted reliable outcomes: higher amounts of biogas (80.22 ± 24.49 NL.kg_SV_^−1^) and methane (47.68 ± 17.55 NL.kg_SV_^−1^) were obtained when implementing a higher amount of olive mill wastewater (30%) (*v*/*v*) in the batch reactors. According to life cycle assessment, the biogas ecoprofile was better when using 20% (*v*/*v*) olive mill wastewater. Similarly, the economic results demonstrated the profitability of the process, with better performances when using 20% (*v*/*v*) olive mill wastewater. These findings confirm the advantages from using farm and food industry by-products for the production of renewable energy as well as organic fertilizers, which could be used in situ to enhance farm sustainability.

## 1. Introduction

The Euro-Mediterranean region boasts an olive oil production exceeding 2950 thousand tons, representing 92.41% of the worldwide production [1] and Italy in its own produced more than 211 thousand tons in 2020. Calabria, in Southern Italy, is the second largest olive oil producer, with over 184,000 ha of olive groves and a production of more than 82,262.7 t of olive oil in 2020 according to ISTAT data [2]. Among the 4480 active mills in Italy, this commodity is mainly produced in small and medium ones with production capacities of up to 500 tons of olive in 76% of cases [3]. This indicates, as for the olive groves, how fragmented the olive processing sector is. Most of these mills adopt three-phase extraction system, generating two kinds of by-products—i.e., olive mill wastewater (OMWW) and olive mill solid waste (OMSW) or olive cake, in addition to olive oil. Messineo et al. [4] estimated that one ton of olives may generate up to 1.6 cubic meters of olive mill wastewater using a three-phase extraction system. This wastewater mainly derives from olive washing as well as from the addition of water during centrifugation. Up until recently, the most common and implemented routines for OMWW management were its storage in evaporation ponds or its controlled disposal on agricultural terrains [5]. However, this practice presents several negative aspects [6]. The concentration of olive milling from both spatial and temporal points of view and the low biodegradability that characterizes OMWW limit its disposal on agricultural lands according to the regulation in vigor and therefore create management problems as well as environmental impacts. Hence, it becomes crucial to look for an eco-friendly way to manage this kind of effluent. In this sense, its recovery for energy production through anaerobic digestion process may constitute a reliable solution.

### 1.1. Application of Anaerobic Codigestion Process to Olive Mill Wastewater

Due to the physical-chemical features that characterize OMWW it is difficult to expect acceptable yields in terms of biogas and methane [7], without applying previous treatments [8].

Bearing in mind the objective of the European Union in terms of energy strategy, which aims at increasing the share of renewable energy up to 32% by 2030 [9], and considering the fact that only 18% of the renewable energy produced in Italy comes from biomass and organic waste, anaerobic codigestion of OMWW with other farm, livestock or food industry by-products constitutes a suitable and a sustainable way to buffer OMWW properties in view of biogas and biomethane production, such as carried out by Kougias et al. [10]. These authors performed batch and continuous trials under mesophilic conditions mixing up to 40% OMWW with swine manure and obtained up to 373 mL.g_VS_ ^−1^ of methane. Battista et al. [11] tested mixtures of olive mill wastewater and olive mill solid waste coming from both three-phase and two-phase extraction systems, with milk whey. They obtained better results (1.23 L_CH4_.Lreactor^−1^.d^−1^) with three-phase solid waste rather than two-phase solid waste. Thanos et al. [12] obtained an increase in biogas yield ranging from 0.7 ± 0.4 L.Lreactor^−1^.d^−1^ to 1.2 ± 0.3 L.Lreactor^−1^.d^−1^ using 40% *v*/*v* of OMWW mixed with liquid pig manure and cheese whey.

### 1.2. Life Cycle Analysis

**Figure 1:**
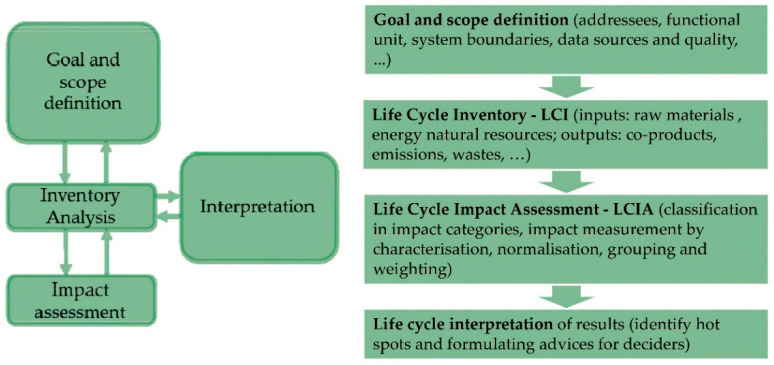
Methodological steps of Life Cycle Assessment (LCA). Source: ISO 14040:2006 [14].

Life cycle-based methodologies are increasingly approved as very powerful and reliable tools to quantify the impact generated from a product/service along the entire production process, which is explored in all its phases and constituents, and throughout its whole duration. In this context, Life Cycle Assessment (LCA) and Life Cycle Costing (LCC) methodologies were developed within the so-called Life Cycle Management (LCM) framework and validated by means of standardization processes. LCA has been defined as “[…] an objective process to evaluate the environmental burdens associated to a product, a process, or an activity by identifying energy and materials usage and environmental releases, and to evaluate opportunities to achieve environmental improvements” [13] and it has been standardized with the International Organization for Standardization (ISO) norms [14,15]. Hence, a correct implementation of the life cycle assessment (LCA), to account for potential direct or indirect loads, considers specific and consecutive steps (Figure 1). The first step aims at defining the goal and scope of the study, the product system and its functions, in terms of specific parameters, such as the Functional Unit (FU), i.e., the measurement unit to which all inputs and outputs data are related, and system boundaries, i.e., the size of the life cycle, which characterize the object of analysis. The description of data quality and allocation procedures is also considered in this step. The second step deals with life cycle inventory (LCI), e.g., a qualitative and quantitative data collection, and then the quantification of incoming and outgoing flows (energy, materials, and emissions) and validation. The life cycle inventory assessment (LCIA) represents the third step and allows relating all data previously considered to specific impact categories, indicators, and characterization models. With the last step, results are interpreted by highlighting potential critical points of the production process and suggesting improvement strategies for production process performances. Over time, LCA has gained increasing interest and many applications have been carried out in different productive sectors and services. Currently, LCA represents the paramount tool to be adopted for achieving environmental certifications, such as carbon footprint, water footprint, and ecological footprint.

In this framework, LCC, representing the alter-ego of LCA for economic analyses, allows considering both the initial and operating costs by suggesting alternatives for optimizing budget allocation during the system/product lifetime [16]. LCC has been developed in the context of management accounting as an investment analysis tool by using the discounting technique and cash flow models, which represent, up to date, the most widespread approaches. However, several methods and standards for performing LCC have grown over time. In this context, environmental LCC assesses internal costs including externalities that are planned to comprise the monetized effects of environmental impacts not directly accounted for in the firms [17]. In these terms, one of the great potentials of life cycle approaches is to properly analyze the whole life cycle of the object under study considering its interactions with the environmental context, upstream and downstream, in terms of supplying inputs, land use, and load or benefit generated by the products, coproducts, by-products or wastes, as well with the economic dimensions, such as production costs, revenues, cash flows, etc. The multiple direct and indirect connections existing between natural contexts and agri-food systems make the latter particularly interesting for sustainability evaluations. Indeed, the accountability of agri-food production processes in generating negative externalities confirms the need for effective tools to quantify environmental impacts consistently with economic analysis, which aims at evaluating firm performances related to cost reduction, income stabilization, productivity, and competitiveness in the markets. From a life cycle perspective, one of the most investigated agricultural systems is olive growing, which is very representative of Mediterranean countries, as well as olive oil industry, which represents, nowadays, a fast-growing sector worldwide [18]. For over ten years, scholars have applied life cycle tools (more or less methodologically integrated) to analyze olive groves by comparing different production systems (traditional vs. innovative; conventional vs. organic), different agricultural areas, different technological solutions (e.g., irrigation systems), with the objective to measure farm sustainability performances. In recent years, the attention of the scientific research has increasingly shifted towards the so-called “circularity evaluation” or, in other terms, the measurement of alternative systems that not only reduce generated environmental impacts, but also make the whole process more efficient by reducing the consumption of raw materials and avoiding waste [19]. In this sense, and in the case of life cycle studies applied to olive oil production, the most challenging direction is to investigate which production methods can represent viable alternatives to optimize a functioning circular economic system by evaluating the way to convert agricultural by-products into energy or into valuable material fractions. For example, Palmieri et al. [20] analyzed the economic and environmental sustainability of an agri-energy chain from pruning residues of olive groves in nine municipalities in southern Italy; Uceda-Rodríguez et al. [21] evaluated the environmental benefits linked to the production of artificial inert materials created with olive pomace as an alternative to the final disposal of this waste in a landfill; Moreno et al. [22] quantified environmental and economic indexes related to different innovative processes of the conversion of biomass coming from olive pruning residues into energy; finally, Batuecasa et al. [23] conducted an LCA of olive oil production by-products by analyzing both anaerobic digestion and conventional disposal on the soil (Table 1).

Considering the above, the present study aims at assessing the production of biogas and biomethane from the codigestion of olive mill wastewater with digestate. Particularly, different percentages of olive mill wastewater in the reactor contents were experimented on under mesophilic conditions in order to evaluate the eventual threshold of using this by-product in the anaerobic codigestion process. In addition, taking into account that each innovative processes should be evaluated in order to verify its economic feasibility and potentially to prevent its impacts or enhance its benefits, this work aims at analyzing the sustainability of the above-mentioned processes by quantifying the environmental loads and economic implications by applying LCA and LCC methodologies in conjunction with specific economic indicators. Therefore, data input was provided by experimental trials carried out in Calabria (Southern Italy). Particularly, global warming, depletion of the ozone layer, eutrophication, acidification, human and ecosystem toxicity, depletion of natural resources, energy consumption, land use, and water use are the environmental impacts categories considered in LCA implementation, while in LCC analysis, operating costs of the production system were accounted for by monetizing inputs and outputs values.

For this purpose, the Material and Methods section provides the methodological approaches used for laboratory experimental trials as well as LCA and LCC methodology implementation. The study outputs are reported in the Results and Discussion section. Finally, suggestions about practical utilization of the study outcomes are reported in the Conclusions.

## 2. Materials and Methods

### 2.1. Biochemical Methane Potential (BMP) of Olive Mill Wastewater

Experimental trials have been conducted at the laboratory scale to assess biochemical methane potential (BMP) of olive wastewater through anaerobic codigestion process.

#### 2.1.1. Anaerobic Codigestion Experiments

Olive mill wastewater (OMWW) was withdrawn during the 2020/2021 campaign from a private mill situated in the Province of Reggio Calabria (Southern Italy, 38°23′28.70″ N; 16°04′31.10″ E), which implements a three-phase extraction system. Digestate (Dig) was withdrawn from a biogas production plant situated in the same province and which already implements olive mill by-products among the feedstock.

**Figure 2:**
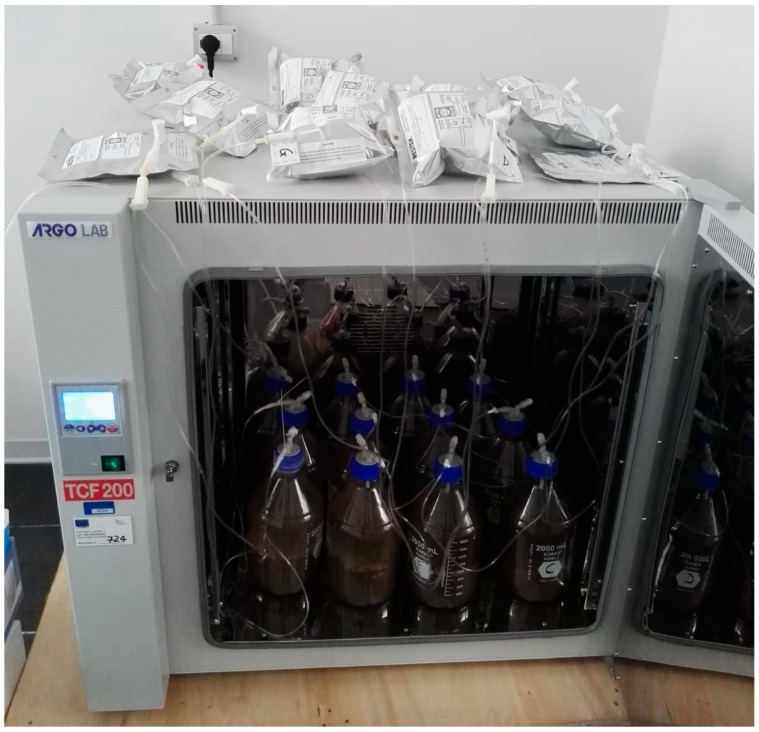
Biochemical methane potential (BMP) tests of olive mill wastewater under mesophilic conditions (37 °C). Source: Picture acquired in our own laboratory.

Biochemical methane potential (BMP) tests were performed using 2000 mL DURAN^®^ GL 45 laboratory glass bottles as reactors. These were later half-filled with mixtures containing 0% (blank), 20% and 30% (*v*/*v*) olive mill wastewater, the remaining content consisted in the digestate. The experimental design is reported in Table 2. Batch reactors were sealed and connected hermetically to the gasbags for biogas sampling. Each thesis was performed in triplicate. Once filled and before sealing, batch reactors were blown through with pure nitrogen (N_2_) to remove atmospheric air at the beginning of the fermentation and favor anaerobic conditions. Then, they were incubated for 30 days at 37 °C in a laboratory forced air oven (AgroLab, Italy, TCF 200) to guarantee mesophilic conditions, as shown in Figure 2.

#### 2.1.2. Substrate and Matrix Characterization

Before each experiment, physical-chemical features of the employed matrices (olive mill wastewater and digestate) as well as the considered mixtures were characterized. This included pH using pH probe (Cryson, GLP 21+), dry content (DC) (%) at 105 °C using a moister analyzer (Ohaus, MB120) and volatile solids VS (%) after ignition at 550 °C using a muffle furnace (Fabber, FBL 70) [24]. Chemical oxygen demand (COD) (g.L^−1^) was measured using a bench photometer after reaction with Hanna high rate COD reagents. In addition, total polyphenols (PPs) were measured for OMWW according to Folin Ciocalteu method [25], total carbon (TC), total nitrogen (TN) and C/N ratio were determined with an elemental analyzer (Leco, CN628).

#### 2.1.3. Biogas Characterization

The produced biogas was sampled in 1 L multilayer foil gas sampling bags (Restek S.r.l.). Biogas content was analyzed using a dual channel micro gas chromatograph (Agilent, 490MicroGC), implementing Molesieve 5A and PoraPLOT Q columns, both running with helium and a micromachined Thermal Conductivity Detector (TCD), while biogas volume was determined according to the water displacement method.

### 2.2. Environmental and Economic Analyses

**Figure 3:**
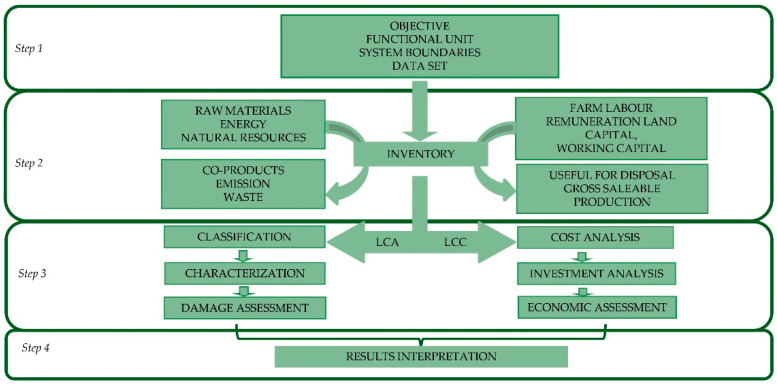
Methodological implementation of Life Cycle Assessment (LCA) and Life Cycle Costing (LCC). Source: Our elaboration.

Environmental and economic analyses of biogas and biomethane production from olive processing by-products through an anaerobic codigestion process were carried out using, respectively, LCA and LCC methodologies. As previously mentioned, LCA follows ISO standards, [14] and [15], which define the principles, framework, and requirements of handling a LCA study. Therefore, the procedure followed in this study includes the following four methodological steps: goal and scope definition; life cycle inventory; life cycle impact assessment (LCIA); and interpretation. The LCC methodology applied in this work was based on the approaches described by Ciroth et al. [26] and Moreau and Weidema [27] and is congruent with and complementary to the LCA methodology. Therefore, the system boundary and the functional unit were similar to those of the LCA (Figure 3). The LCC was also implemented in conjunction with specific economic indicators to assess the economic profitability of biogas production.

#### 2.2.1. Scenarios Description

The production of biogas from anaerobic codigestion was evaluated both from environmental and economic points of view, considering the mixtures previously tested at the laboratory scale. Hence, a scaling up from the laboratory level to the industrial level was performed. Specifically, an anaerobic reactor that produces biogas and generates an electrical power of 200 kW was considered for the analyses.

Environmental and economic impact assessments were then performed considering only the mixtures containing OMWW (the control thesis was excluded). Particularly, the following was evaluated: biogas production from the mix containing 20% (*v*/*v*) OMWW and 80% (*v*/*v*) digestate with a retention time of 16 days (Thesis 2); biogas production from the mix containing 30% (*v*/*v*) OMWW and 70% (*v*/*v*) digestate with a retention time of 29 days (Thesis 3).

The scaling up was based on the results obtained in the laboratory experiments, modelling the size of the plant into an industrial one considering matrix availability. It has been assumed that the plant is located in the vicinity of another anaerobic digestion plant with an electrical power of 998 kW that produces an annual quantity of digestate equal to 37,000 t, enough to satisfy the feeding needs of the plant fed with OMWW, which, according to the different retention times, requires annual quantities of matrices equal to 45,625.00 (Thesis 2) and 25,172.41 t (Thesis 3).

Specific assumptions for environmental and economic assessments are discussed in the following paragraph.

#### 2.2.2. Goal and Scope Definition, Functional Unit and System Boundaries

**Figure 4:**
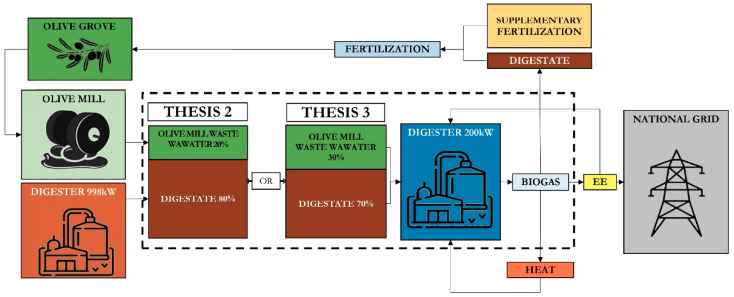
Flowchart of the system boundaries considered in the two scenarios. Source: Our elaboration.

For both LCA and LCC, the same assumptions were used for life cycle modelling so that the life cycle of biogas production from OMWW could be assessed according to common criteria from both environmental and economic perspectives. In particular, the function to be analyzed is OMWW recovery to energy; therefore, “1 m^3^ of normalized biogas” has been defined as the Functional Unit (FU). Since wastewater is normally considered a waste product of the olive milling process, it has been chosen to limit the system boundaries “from digester gate to the biogas production”, considering OMWW as a residual product with zero impact (Figure 4).

#### 2.2.3. Specific LCA Implementation

Data on quantities of matrices, transport, quantities of biogas and generated heat as well as produced digestate were taken from laboratory trials and scaled up to the 200 kW electrical power plant. According to current regulatory requirements, the plant electricity consumption has been set at 11% for self-consumption. Secondary data on fuel production for transport and digester construction were taken from the Ecoinvent 3.5 database. The methane and ammonia emissions from digestate storage were assessed according to Lovarelli et al. [28]. Fugitive methane losses from digesters and post-digesters and losses during biogas treatment and combustion were considered equal to 2% following Dressler et al. [29].

The inventory data (Table 3) were processed through Simapro 8.5 software using the ILCD 2011 midpoint impact assessment method [30], through which the following impact categories were assessed: climate change; ozone depletion; human toxicity, non cancer effects; human toxicity, cancer effects; particulate matter; ionizing radiation HH; ionizing radiation E (interim); photochemical ozone formation; acidification; terrestrial eutrophication; freshwater eutrophication; marine eutrophication; freshwater ecotoxicity; land use; water resource depletion; mineral, fossil and ren resource depletion.

Cut-off criteria were set ignoring all inventory data that would have an impact of less than 1%, such as energy for plant control computers.

The main limitation of the study lies in the scaling operation, whereby productions are directly proportional to those obtained in laboratory trials. Since a decrease in plant efficiency at full-scale is possible, a sensitivity analysis was carried out, reducing the biogas production by 10% and 20%.

#### 2.2.4. Specific LCC Implementation and Profitability Analysis

The LCC analysis aimed at evaluating the overall cost of the two scenarios of biogas production under study (Thesis 2 and Thesis 3). Data collection was conducted in parallel with the inventory phase of LCA to estimate costs related to plant acquisition, operation, and disposal in accordance with Gonzalez et al. [31]. As pointed out by Herbes et al. [32], the site-specific conditions in which the process takes place should be considered. Therefore, in performing the economic analysis, site-specific cost drivers are taken into account.

The initial investment cost for the plant acquisition was EUR 900,000 according to the current market prices, corresponding to a specific cost of EUR 4500 per kW.

Operating costs were split into three categories: materials and services, labor, quota, and other attributions. In the first category, only transport costs for matrix handling were considered, assuming an average distance of 500 km per year. The diesel average price was taken as EUR 0.92 per liter, taking into account an average consumption of 0.05 L.t^−1^.km^−1^.

In this work, the purchase price of both OMWW and digestate was assumed to be EUR 0 per ton. In the first case, we assumed that the transport cost is covered by olive mills, which avoid the traditional disposal of the wastes on the soil. In the second case, the cost of digestate was considered for free.

Within the labor costs, human labor cost based on local current wage (EUR 8 per hour) and administrative overheads (EUR 10.3 per hour) were included.

In the quota and other attributions category, all those cost items not directly attributable to specific biogas production process stage, represented by quotas (i.e., depreciation, maintenance and insurance), interests in advance capital and capital goods, land rent and levies, were considered.

The expected revenues were estimated considering only the sale of electricity after internal consumption. The electricity produced was assumed to be fed into the national grid. A FiT tariff of EUR 0.233 per kWh was considered [33].

Estimation of end of life costs for the biogas plant disposal was obtained from the literature [31]. The plant disposal was estimated by subtracting from disposal cost the used equipment revenue.

The following assumptions were made to carry out the economic analysis of the two scenarios:

- All of the costs and revenues were discounted for the entire life cycle of 15 years (plant lifetime).
- To select a discount rate, the opportunity cost approach in terms of alternative investments with similar risks and times was used [34]. Here, a discount rate set to 5% was assumed, as in other studies [31,35].
- Constant prices by excluding adjustments for inflation [36] were taken into account.

In order to evaluate the investment feasibility of the biogas production scenarios, specific economic indicators were identified—i.e., Discounted Gross Margin (DGM), Net Present Value (NPV), Internal Rate of Return (IRR) and Discounted Payback Period (DPP). These represent the most common indicators used to compare investment options, which are based on the cash flow model [32].

The DGM indicator provides information on project profitability, as advised by Mel et al. [37] and Stillitano et al. [38], defined in Equation (1):

(1) $$\mathrm{DGM}=\overset{n}{\underset{t=1}{\sum }}\frac{{\mathrm{TR}}_{t}}{{\left(1+r\right)}^{t}}-\frac{{\mathrm{VC}}_{t}}{{\left(1+r\right)}^{t}}$$

where TR_t_ is the total revenue in the t-th year; VC_t_ is the variable cost in the t-th year; t is the time of the cash flow (year); n is the plant lifetime (15 years) and r is the discount rate (5%).

The NPV and IRR indicators were calculated according to Equations (2) and (3), respectively, as suggested by Moreno et al. [39]:

(2) $$\mathrm{NPV}=\overset{n}{\underset{t=1}{\sum }}\frac{{\mathrm{CF}}_{t}}{{\left(1+r\right)}^{t}}{-\;I}_{0}$$

where t is the time of the cash flow (year); n is the plant lifetime (15 years); CF_t_ is the net cash flow in the t-th year; r is the discount rate (5%) and I_0_ is the initial investment, which equals the total facility investment.

(3) $$\overset{n}{\underset{t=1}{\sum }}\frac{{\mathrm{CF}}_{t}}{{\left(1+\mathrm{IRR}\right)}^{t}}-I_{0}=0$$

where IRR is the discount rate, which will make the NPV equal to zero.

When the conditions NPV > 0 and IRR > r occur, the investment is profitable; otherwise, it should be rejected [40].

The formula for calculating the DPP indicator is presented in Equation (4), as suggested by Tse et al. [41]:

(4) $$\mathrm{DPP}=\mathrm{LNC}\frac{\mathrm{ADC}}{\mathrm{DCA}}$$

where LNC is the last period with a negative discount cumulative cash flow; ADC is the absolute value of discount cumulative cash flow at the end of the period LNC; DCA is the discount cash flow during the period after LNC.

As argued by Ong and Chun [42], the payback period, defined as the expected number of years required to recover the initial investment, is often used as an indicator of a project’s riskiness. In any case, the payback period must be shorter than the time horizon considered.

Lastly, each indicator value has been defined for the FU of 1 m^3^ of normalized biogas.

As a final step, a sensitivity analysis was performed for the two scenarios to examine the influence of varying specific parameters over the economic indicators under study [43]. The variables independently evaluated were discount rate (r) set to be floated with ± 20% and biogas yields floated with −10% and −20%.

## 3. Results and Discussion

### 3.1. Results of Biochemical Methane Potential (BMP) of Olive Mill Wastewater

#### 3.1.1. Matrix and Substrate Characterization

The results of the initial characterization of the matrices and the substrates are, respectively, reported in Table 4.

According to the obtained data, the pH value of OMWW is, as expected, very low and similar to values reported by other authors [44], while values inherent to theses subjected to anaerobic digestion process are between 6.93 ± 0.03 and 7.97 ± 0.16, with optimal values for both mixes, meaning that the comatrix, i.e., the digestate, exerted a good buffering effect. Dry content (DC) in all cases does not exceed 10%, indicating that the process runs in wet conditions, which consists of the operating mode of most of large-scale reactors worldwide [45,46]. Volatile solid (VS) or organic substance content also represents an important parameter for the anaerobic digestion process as it refers to the susceptible content to be decomposed [47]. In addition, the chemical oxygen demand (COD), whose values are between 70.35 ± 4.47 and 80.82 ± 1.59 g.L^−1^ for the substrates subjected to AcoD, measures the content of oxidizable compounds in the substrate [48] and theoretically enable predicting methane production as 1 g of converted COD corresponds to a maximum of 350 mL of methane [49]. In the three theses, the carbon/nitrogen ratio (C/N) was below the recommended value that should be comprised between 20 and 30, with an optimal value of 30. However, Guarino et al. [50] investigated the effect of a wider C/N interval ranging from 9 to 50 on anaerobic digestion of buffalo manure and obtained a high biomethane productivity (around 60–70%) even with lower values than those obtained in our experiments. C/N values decreased in favor of nitrogen as OMWW content in the reactor increased.

As the trials aim at assessing the BMP of the OMWW, it was important to quantify polyphenol (PP) contents since they represent inhibiting compounds of the bacterial pool, particularly methanogens. The analysis revealed an amount of 4.60 g.L^−1^.

#### 3.1.2. Biogas and Methane Yields

**Figure 5:**
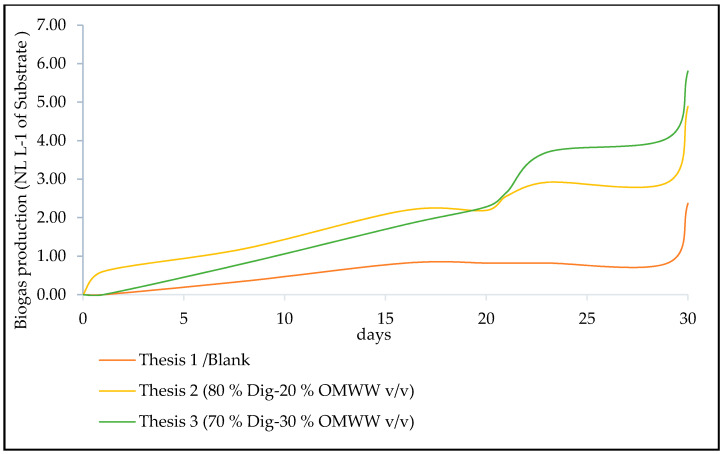
Cumulative biogas production for 30-day AcoD of olive mill wastewater. Values are the mean production values obtained from the three replicates of each thesis at different sampling time. Source: Our elaboration.

The biogas volume recorded in each sampling date as well as methane content were normalized to normal liters (dry gas, at temperature = 0 °C and pressure = 1013 hPa), according to the standard procedures described in the VDI 4630 [48], as carried out in [44]. Cumulative biogas production of the tested theses during the AcoD period is represented in Figure 5. Higher biogas production was registered in Thesis 2, which contains 20% *v/v* olive mill wastewater, until day 20. After that, this tendency changed in favor of Thesis 3, which registered a total amount of biogas equal to 5.80 ± 1.77 NL.L^−1^ of substrate, corresponding to 80.22 ± 24.49 NL.kg_SV_^−1^.

**Figure 6:**
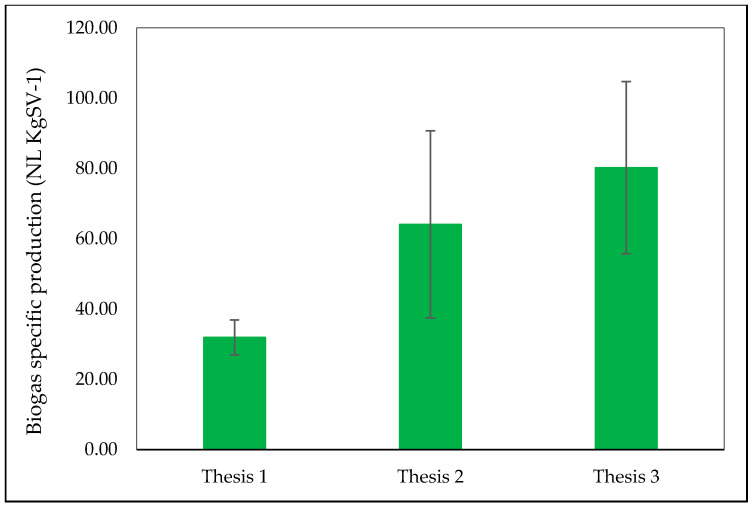
Mean values ± St. Dev. of total biogas specific production for 30 days AcoD of olive mill wastewater. Source: Our elaboration.

Considering biogas total amount, Thesis 2 (with 4.88 ± 2.03 NL.L^−1^ of substrate corresponding to 64.06 ± 26.64 NL.kg_SV_^−1^) and Thesis 3, respectively, recorded 2- and 2.5-times higher productions than that of the blank equal to 2.37 ± 0.37 NL.L^−1^ of substrate corresponding to 31.89 ± 4.98 NL.kg_SV_^−1^ (Figure 6), meaning that reactor content in OMWW favored biogas production. Nevertheless, statistical analysis by performing one-way ANOVA did not show any significant difference (F = 4.082; df (2; 6), Pr (>F) = 0.076).

**Figure 7:**
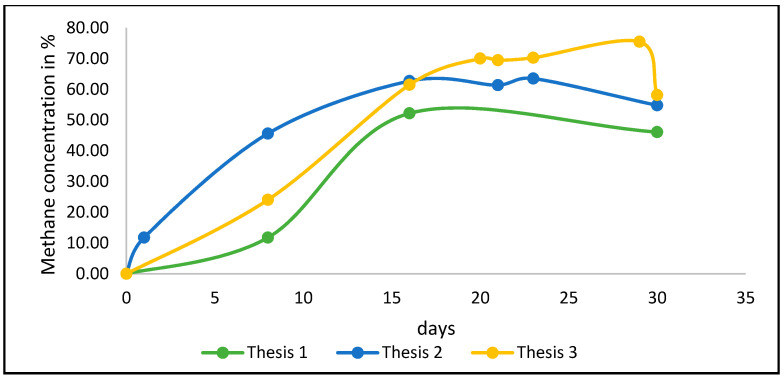
Methane content in the biogas expressed as percentage. Source: Our elaboration.

**Figure 8:**
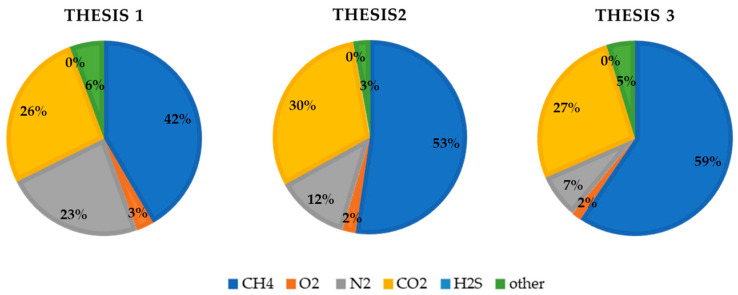
Biogas composition considering the whole process of OMWW AcoD. Source: Our elaboration.

Regarding biogas composition, the highest methane percentage, 75.48%, was obtained by Thesis 3. Methane content had the same tendency as biogas production, with higher amount in Thesis 2 at the beginning of AcoD process until the 16th day, after which a decline was observed in both Theses 1 and 2, whereas higher amounts were found in Thesis 3 until day 29, after which they decreased by 58.12% (Figure 7). Total amount of methane during the whole process was equal to 0.99 ± 0.06 NL.L^−1^ of substrate, corresponding to 13.29 ± 0.79 NL.kg_SV_^−1^, 2.55 ± 1.15 NL.L^−1^ of substrate, corresponding to 33.53 ± 15.15 NL.kg_SV_^−1^, and 3.45 ± 1.27 NL.L^−1^ of substrate, corresponding to 47.68 ± 17.55 NL.kg_SV_^−1^, respectively, for Theses 1, 2 and 3. Additionally, for methane content, no significant difference was found (F = 4.997; df (2; 6), Pr (>F) = 0.0528). Figure 8 illustrates the biogas composition considering the overall process for the three tested theses.

Biogas and biomethane results obtained in this study are lower than those reported by other authors, who implemented OMWW in an anaerobic codigestion process. Indeed, experimental trials performed by Zema et al. [44] provided 0.362 Nm^3^.kg_TVS_^–1^ of biogas (0.187 Nm^3^.kg_TVS_^–1^ of methane) and 0.176 Nm^3^ kg_TVS_^–1^ of biogas (0.067 Nm^3^.kg_TVS_^–1^ of methane) after 25 days using, respectively, 20% and 30% OMWW with a polyphenol concentration of 2.8 g.kg^−1^ in a mesophilic AcoD process with digested liquid manure. In contrast with the results presented here, they yielded higher amounts of biogas and methane with lower quantities of OMWW. Bovina et al. [51] reported an increase in biogas and methane yield with the increasing of OMWW content instead of sewage sludge, obtaining the best performances with 25% OMWW (with 1.01 ± 40 g.L^−1^ of polyphenols)—i.e., 116 NL.kg_VS_^–1^ of methane. Calabrò et al. [52] used raw and concentrated OMWW with polyphenols values ranging between 1.1 ± 0.12 and 4.4 ± 0.03 g.L^−1^ in order to obtain up to 2 g.L^−1^ PPs in the blends they tested in batch under mesophilic conditions. They obtained 0.419 NL.g_TVS_^–1^ with a PP concentration of 0.5 g.L^−1^. The blend with 2 g.L^−1^ PPs provided better results due to the adaptation of the inoculum to polyphenols (170 against 45 NL.g_TVS_^–1^ when using non acclimated inoculum).

Accordingly, it can be stated that the lower amounts obtained in this study are mainly due to the high polyphenol (PP) contents, equal to 4.60 g.L^−1^. Regarding this aspect, Borja et al. [53] and Fedorak et al. [54] suggest not exceeding a phenol concentration of 2 g.L^−1^ to avoid an inhibiting effect on the methanation process.

### 3.2. Environmental Results

Environmental impacts related to the production of 1 m^3^ of biogas are presented in Table 5. The ecoprofile of biogas from “Thesis 2” shows better results than “Thesis 3”; however, they are fully comparable, due to the higher biogas production that could be achieved with “Thesis 2” considering an annual duration, as it has shorter retention times and therefore allows more matrices to be processed. The shorter retention times can be attributed to the lower amount of OMWW in the mix, which favors a faster start-up of the anaerobic digestion and methanation processes.

The impacts are coherent with those obtained in other works such as [28,55,56], but in order to compare them, some clarifications are needed. Indeed, the above-mentioned studies used the production of electricity from cogeneration as a functional unit, whereas in the present study we have limited the study to the production of biogas. Therefore, the impacts of cogeneration should be added to the impacts presented in Table 4 and should be attributed to the production of electricity. The impacts could be even more favorable if the avoided emissions from the storage of input digestate and wastewater had been considered in the calculation of emissions. In this regard, the emissions from storage could be considered zero due to the balance between avoided and generated emissions. In addition, the assumptions made by Lovarelli et al. [28] were used to estimate ammonia and methane emissions from digestate management, although they refer to digestate from livestock manure. In the case of the two tested theses, the mixes are largely made from digestate, so the experimented anaerobic digestion process allows the recovery of all biogas still obtainable from this matrix, leaving, as an output, a rather exhausted “second generation” digestate.

Therefore, methane emission resulting from the storage of digestate could also be considered zero, which together with the avoided impacts for the management of the incoming digestate could generate a strong reduction in impacts.

**Figure 9:**
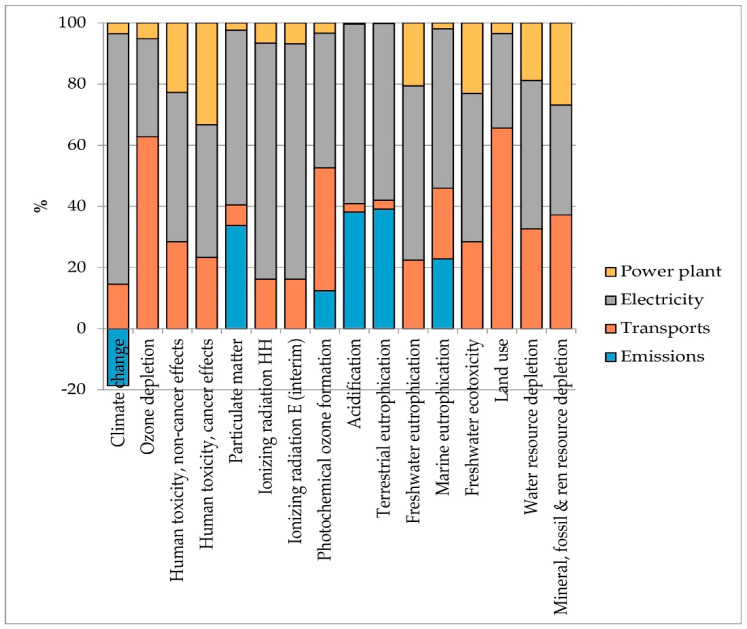
Contribution analysis in Thesis 2. Source: Our elaboration.

**Figure 10:**
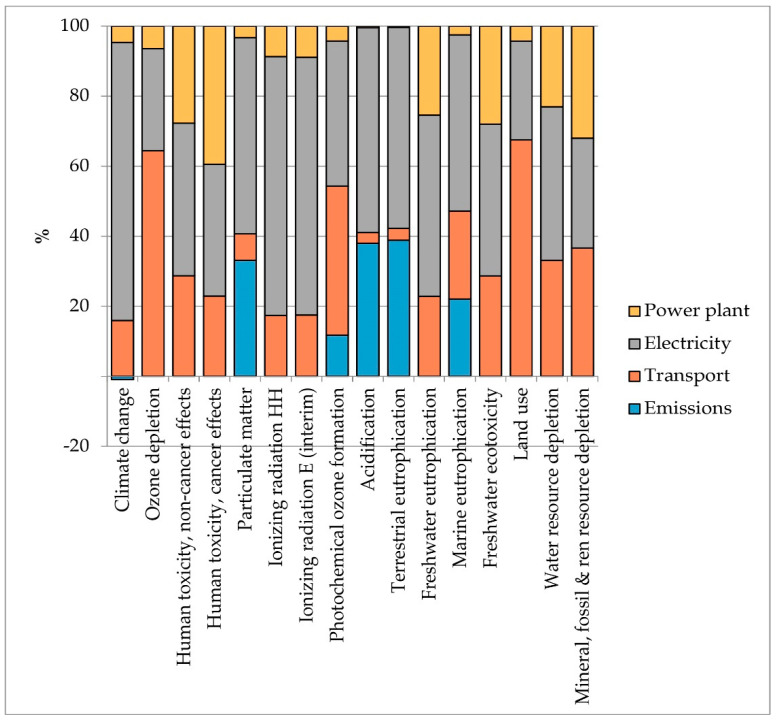
Contribution analysis in Thesis 3. Source: Our elaboration.

The analysis of the contribution of individual processes was carried out for both theses and it is shown in Figure 9 and Figure 10. It emerged that the electricity is the main hotspot in both analyzed scenarios and for all impact categories. Electricity has been considered as an external input but it is modelled on the basis of 11% legally required self-consumption. The use of electricity as an input purchased on the market is a consequence of the system boundaries being set for biogas production and not for the combined production of heat and power (CHP). Using self-produced energy would probably result in significantly lower impacts. Transport is, on average, the second hotspot. The impacts of this process are related to the movement of matrices. This element is a critical factor in the production of biogas from agricultural waste since the need for matrices to feed the plant often requires them to be supplied from long distances. In the specific case study, the amount of wastewater needed to operate the plant for one year is generated from about 1100 ha (11 km^2^) of olive grove, while the digestate is all produced by a single 998 kW anaerobic digestion plant. Therefore, assuming that the OMWW digestion plant is built close to the 998 kW plant, transport is only related to wastewater and therefore an average supply distance of 5 km has been estimated.

The positive impacts in the Climate Change category are due to the fixation of CO_2_ during the anaerobic digestion process. In Thesis 2, the balance between fixation and emissions is positive, while in Thesis 3, emissions almost equal CO_2_ fixation, so the positive impact of this phenomenon is almost reduced to zero. The impacts from the plant refer to a plant of 200 kW electrical power with a lifespan of 15 years.

Since the scaling up operation could lead to lower yields than those found in the laboratory experiments, due to a lower control of the anaerobic digestion process caused by the full-scale dimensions of the 200 kW plant, the effect of a reduction in the production capacity of the two theses was analyzed, assuming yield decreases of 10% and 20%, respectively, in both tested theses. The results of the sensitivity analysis show a linear increase in impacts in almost all impact categories (on average +6% in the hypothesis of a 10% yield reduction and +13% in the hypothesis of a 20% yield reduction), except for the Climate Change category. Impacts in terms of GHG emissions increase exponentially (+137.68% and +243.03% for Thesis 2; +103.12% and +112.79% for Thesis 3) and these results are attributed to the lower production efficiency linked to the use of inputs. Indeed, for the same quantity of implemented inputs, the sensitivity scenarios predict lower yields; therefore, the incidence of impacts per unit of product increases (Table 6). Given the high influence of electricity use on Climate Change impacts (see Figure 9 and Figure 10), a reduction in yields has very clear consequences on this impact category. Thesis 2 is more sensitive than Thesis 3 as the amounts of implemented inputs in the process are larger given the shorter retention times in Thesis 2.

Different modelling of the two production processes with self-consumption of the energy produced instead of energy purchased on the market could lead to a flattening of the effects of yield reductions on climate change.

The methane yield of the two tested theses is in favor of Thesis 3, which had an average peak of 68.6% compared to 61.2% for Thesis 2. Extending the boundaries of the system to cogeneration and using the amount of energy produced from biogas, the results could change in favor of Thesis 3.

### 3.3. Economic Results

The main results of the economic evaluation are presented in Table 7. It is worth noting that the findings are clearly influenced by the biogas production yield, which was greater in Thesis 2 than in Thesis 3 considering an annual duration. Therefore, the best scenario in terms of total life cycle cost was Thesis 2, with a value of EUR 4.55 per m^3^ per year vs. EUR 6.94 per m^3^ per year (achieved by the Thesis 3). In both scenarios, the cost driver was initial investment, contributing, in overall, with 88.8% of the total LCC.

The analysis of operating costs showed that, in terms of FU, the quota and other attributions category was the greatest contributor to the total operating costs (89.8%). This is due to the higher costs of maintenance and depreciation incurred for plant investment.

Within the material and services category, only transport cost for matrix handling was included and was estimated at 9% of the total operating cost in both scenarios. Since we assumed that the supply of raw materials was free (see Section 2.2.4), no raw material cost was calculated.

The results obtained from the feasibility analysis of the two scenarios under study are shown in Table 8. The findings indicated that both scenarios were profitable. In fact, under assumptions considered for each economic indicator, it was found that:

- NPV was greater than zero, being EUR 0.37 per m^3^ and EUR 0.20 per m^3^ for Thesis 2 and Thesis 3, respectively;
- IRR was higher than discount rate, being 21.64% and 11.71% for Thesis 2 and Thesis 3, respectively;
- DPP was shorter than the time horizon considered, being 5.05 and 8.62 years for Thesis 2 and Thesis 3, respectively.

However, the Thesis 2 showed the best performance in most of the examined indicators. This is largely due to the bigger revenues, which were estimated considering only the sale of electricity after internal consumption, in Thesis 2 compared to Thesis 3.

**Figure 11:**
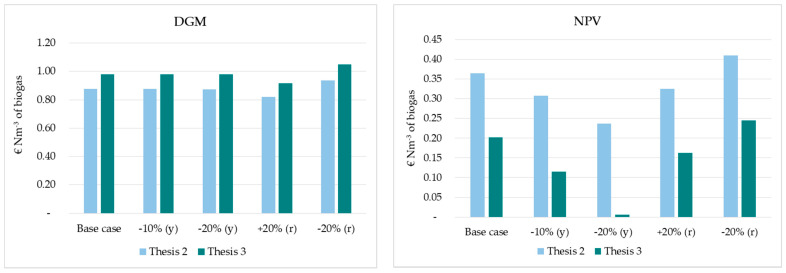

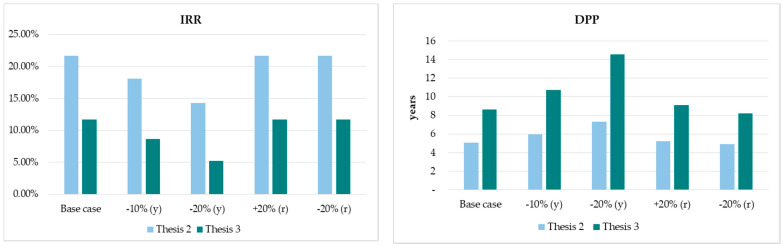
Sensitivity analysis for the two scenarios under study: −10% and −20% represent a decrease in biogas yield (y); +20 and −20% represent, respectively, an increase and decrease in discount rate (r) (DGM = Discounted Gross Margin; NPV = Net Present Value; IRR = Internal Rate of Return; DPP = Discounted Payback Period). Source: Our elaboration.

Figure 11 shows the sensitivity analysis carried out by changing the discount rate with a ±20% variation and biogas yield floated with −10% and −20%. The results indicated the biogas yield was the most important parameter in the profitability variation. This factor had a remarkable impact on NPV, IRR and DPP indicators. Its decrease led to the worst economic configuration of the plant in both scenarios, and has shown the following:

- Considering a −10% variation in biogas yield, −15.61%, −16.56% and +18.44% variations were observed in the NPV, IRR and DPP, respectively, for Thesis 2, and −43.03%, −26.57% and 24.64% variations for Thesis 3.
- Considering a −20% variation in biogas yield, −35.11%, −33.95% and 45.43% variations were observed in the NPV, IRR and DPP, respectively, for the Thesis 2, and −96.81%, −55.67% and 68.75% variations for the Thesis 3.

These findings were consistent with results reported by Li et al. [43]. Less significant variations were achieved for the DGM indicator, ranging from −0.04% for Thesis 2 to −0.05% for Thesis 3, decreasing the biogas yield by 10%, and −0.08% and −0.11%, respectively, with a decrease of 20%.

The sensitivity results also showed that changes in the discount rate affect the magnitude of NPV in both scenarios, in accordance with the studies by Herbes et al. [32] and Hamedani et al. [57]. NPV decreased by more than 11% for Thesis 2 and 19% for Thesis 3 when the discount rate increased by 20%. When the discount rate decreased by 20%, NPVs rose accordingly by 12.24% in Thesis 2 and 21.25% in Thesis 3. While no change was recorded for the IRR indicator, weak changes were achieved for DGM and DPP.

## 4. Conclusions

The present study illustrates the intermediate results conducted in the framework of the Sustainability of the Olive oil System (S.O.S.) project, with a particular interest in the recovery of olive mill wastes for energy purposes. The findings up to now considering technical, economic, and environmental aspects are very promising, since laboratory experimental trials showed higher amount of biogas (80.22 ± 24.49 NL.kg_SV_^−1^) and methane (47.68 ± 17.55 NL.kg_SV_^−1^) when implementing a higher content of olive mill wastewater (30% of *v*/*v* in our case). Our results are lower than those obtained in other studies, due to the high content of OMWW in polyphenols; however, they suggest the possibility to explore furthermore AcoD of OMWW considering other farm, livestock, or food industry by-products to enhance biogas and biomethane production, and consequently increase the positive effects on the environment by limiting the impacts, as supported by LCA. In this regard, the analysis of environmental impacts has shown that the production of biogas from OMWW is advantageous from several points of view. In fact, through anaerobic digestion, multiple benefits are obtained such as: the management of a critical waste to be managed from an environmental point of view; the valorization of the biogas present in the digestate that, otherwise, would be dispersed in the environment, representing an environmental load; the production of renewable energy produced exclusively from waste; the production of an exhausted digestate that can be used as organic fertilizer and avoid the impacts generated by the production and distribution of synthetic fertilizers. This represents a strategy for implementing circular economy models, with benefits that go beyond just improving environmental performances. In fact, the economic analysis has demonstrated the profitability of this solution, which, due to the feed-in tariff guaranteed for 15 years, allows dealing with the investment for the plant without the risk of the aleatory energy selling price. Moreover, the recovery and valorization of thermic energy for district heating would allow a further advantage, both for the producer and for the community—using a source of heating with a low environmental impact and that is economic and independent from the public network and therefore free from fixed costs in the bill. Current policies and subsidies encourage the use of by-products instead of energy crops for renewable energy production, so this could be a further stimulus to the spread of small plants dedicated to the digestion of OMWW. In addition, global policies now focus on the adoption of sustainable and circular development models, so it is desirable that the force research should be concentrated on the valorization of waste, transforming it into by-products capable of producing benefits for the whole community.

## Figures and Tables

**Table 1:** Analysis of the main literature dealing with life cycle studies applied to agricultural by-products recovery. Source: Our elaboration.

Authors	Year	Title	Journal	Field of Application	Applied Methodologies
Palmieri, N., Suardi, A., Alfano, V., Pari, L.	2020	Circular Economy Model: Insights from a Case Study in South Italy.	*Sustainability*	Electricity production from pruning residues of olive groves	Profitability and efficiency ratios; Greenhouse gas emissions
Uceda-Rodríguez, M., López-García, A.,B., Moreno-Maroto, J.,M., Cobo-Ceacero, C., J., Cotes-Palomino, M.,T., Martínez García, C.	2020	Evaluation of the Environmental Benefits Associated with the Addition of Olive Pomace in the Manufacture of Lightweight Aggregates.	*Materials*	Olive pomace recycling as a substitute for clay	Life Cycle Assessment
Moreno, V.C., Iervolino, G., Tugnoli, A., Cozzani, V.	2020	Techno-economic and environmental sustainability of biomass waste conversion based on thermocatalytic reforming.	*Waste Management*	Biomass waste (olive wood pruning and digestate) to energy conversion process	Mass and energy balances
Batuecasa, E., Tommasi, T., Battista, F., Negro, V., Sonetti, G.	2019	Life Cycle Assessment of waste disposal from olive oil produion: Anaerobic digestion and conventional disposal on soil.	*Journal of Environmental Management*	Management of by-products from olive oil production: solid–liquid olive pomace and olive mill wastewater	Life Cycle Assessment

**Table 2:** Experimental setup of biochemical methane potential (BMP) tests. Source: Our elaboration.

	Thesis 1 (Blank)	Thesis 2	Thesis 3
Olive mill wastewater content (*v*/*v*)	0%	20%	30%
Digestate (*v*/*v*)	100%	80%	70%

**Table 3:** Inventory data. Source: Our elaboration.

	Unit	Thesis 2	Thesis 3
Products			
Biogas	m^3^	1.00	1.00
Primary inputs			
Carbon Dioxide	g	428.40	417.69
Inputs			
Transports	t.km^−^^1^	0.20	0.26
Electricity	kWh	0.67	0.75
Power plant	p	2.24 × 10^−7^	3.42 × 10^−7^
Emissions			
Carbon dioxide	g	71.97	71.97
Methane	g	12.23	13.71
Ammonia	g	1.41	1.58
Heat	MJ	0.52	0.52

**Table 4:** Matrix and substrate preliminary characterization. Values are expressed as mean ± St. Dev of minimum three replicates for each parameter and each matrix/substrate. Source: Our elaboration.

	Unit	OMWW	Dig/Blank	Thesis 2	Thesis 3
pH		4.65 ± 0.05	7.97 ± 0.16	7.20 ± 0.01	6.93 ± 0.03
DC	%	8.18 ± 0.15	9.31 ± 0.52	9.46 ± 0.74	8.99 ± 0.56
VS _dry matter_	%	82.08 ± 0.34	79.84 ± 0.72	80.59 ± 0.12	80.47 ± 0.42
COD	g.L^−1^	125.39 ± 3.57	70.35 ± 4.47	80.82 ± 1.59	79.56 ± 1.27
TC	g.kg^−1^	/	481.57 ± 0.77	487.53 ± 3.15	491.13 ± 2.40
TN	g.kg^−1^	/	26.65 ± 0.48	27.83 ± 0.11	29.84 ± 0.27
C/N		/	18.08 ± 0.30	17.52 ± 0.17	16.46 ± 0.20
PPs	g.L^−1^	4.60	/	/	/

**Table 5:** Characterization of impacts linked to 1 m^3^ of biogas production. Source: Our elaboration.

Impact Categories	Unit	Thesis 2	Thesis 3
Climate change	kg CO_2_ eq	2.22 × 10^−^^1^	3.12 × 10^−^^1^
Ozone depletion	kg CFC-11 eq	1.02 × 10^−^^8^	1.26 × 10^−^^8^
Human toxicity, noncancer effects	CTUh	2.10 × 10^−^^8^	2.63 × 10^−^^8^
Human toxicity, cancer effects	CTUh	9.25 × 10^−^^9^	1.19 × 10^−^^8^
Particulate matter	kg PM2.5 eq	2.78 × 10^−^^4^	3.17 × 10^−^^4^
Ionizing radiation HH	kBq U235 eq	2.14 × 10^−^^2^	2.51 × 10^−^^2^
Ionizing radiation E (interim)	CTUe	6.51 × 10^−^^8^	7.62 × 10^−^^8^
Photochemical ozone formation	kg NMVOC eq	9.89 × 10^−^^4^	1.18 × 10^−^^3^
Acidification	molc H+ eq	1.11 × 10^−^^2^	1.25 × 10^−^^2^
Terrestrial eutrophication	molc N eq	4.86 × 10^−^^2^	5.48 × 10^−^^2^
Freshwater eutrophication	kg P eq	1.49 × 10^−^^5^	1.84 × 10^−^^5^
Marine eutrophication	kg N eq	5.67 × 10^−^^4^	6.59 × 10^−^^4^
Freshwater ecotoxicity	CTUe	4.64 × 10^−^^1^	5.83 × 10^−^^1^
Land use	kg C deficit	1.41 × 10^−^^1^	1.73 × 10^−^^1^
Water resource depletion	m3 water eq	1.43 × 10^−^^4^	1.78 × 10^−^^4^
Mineral, fossil and ren resource depletion	kg Sb eq	2.63 × 10^−^^6^	3.37 × 10^−^^6^

**Table 6:** Sensitivity analysis of results with reductions in biogas yield, respectively, of −10% and −20%. Impact deviations from the baseline scenario. Source: Our elaboration.

	Thesis 2		Thesis 3	
Impact category	−10%	−20%	−10%	−20%
Climate change	+137.68%	+243.03%	+103.12%	+112.79%
Ozone depletion	+7.55%	+16.99%	+7.87%	+17.70%
Human toxicity, noncancer effects	+5.69%	+12.81%	+6.26%	+14.09%
Human toxicity, cancer effects	+6.30%	+14.18%	+6.93%	+15.59%
Particulate matter	+4.78%	+10.75%	+4.89%	+10.99%
Ionizing radiation HH	+2.54%	+5.72%	+2.91%	+6.54%
Ionizing radiation E (interim)	+2.56%	+5.76%	+2.93%	+6.59%
Photochemical ozone formation	+11.02%	+17.83%	+11.03%	+18.25%
Acidification	+4.58%	+10.32%	+4.62%	+10.38%
Terrestrial eutrophication	+4.70%	+10.57%	+4.73%	+10.63%
Freshwater eutrophication	+4.77%	+10.73%	+5.36%	+12.06%
Marine eutrophication	+5.31%	+11.96%	+5.51%	+12.41%
Freshwater ecotoxicity	+5.72%	+12.88%	+6.30%	+14.17%
Land use	+7.68%	+17.29%	+7.98%	+17.96%
Water resource depletion	+5.71%	+12.85%	+6.24%	+14.05%
Mineral, fossil and ren resource depletion	+7.12%	+16.02%	+7.62%	+17.14%

**Table 7:** Life cycle costs of the biogas plant under two scenarios (EUR.m^−3^.year^−1^ of biogas). Source: Our elaboration.

Cost Item	Thesis 2	Thesis 3
Initial investment cost	4.04	6.16
Operating costs	0.34	0.53
-Materials and Services	0.004	0.01
-Labor	0.03	0.05
-Quotas and other duties	0.31	0.47
End of life disposal costs	0.17	0.25

**Table 8:** Comparison of the economic feasibility for the two scenarios under study. Source: Our elaboration.

Economic Indicator	Unit	Thesis 2	Thesis 3
Discounted Gross Margin (DGM)	EUR.m^−3^	0.88	0.98
Net Present Value (NPV)	EUR.m^−3^	0.37	0.20
Internal Rate of Return (IRR)	%	21.64	11.71
Discounted Payback Period (DPP)	years	5.05	8.62

## Data Availability

Data is contained within the article. Raw data of the study results may be available on request from the corresponding author.

## Abbreviations

%	percent
°C	degree Celsius
AcoD	anaerobic codigestion
BMP	biochemical methane potential
C/N	carbon/nitrogen ratio
CHP	combined heat and power
COD	chemical oxygen demand
DC	dry content
DGM	discounted gross margin
Dig	digestate
DPP	discounted payback period
E	Ecosystem
EUR	Euro
EUR.m^−3^	Euro per cubic meter
EUR.m^−3^.year^−1^	Euro per cubic meter per year
FU	functional unit
g.kg^−1^	gram per kilogram
g.L^−1^	gram per liter
g	gram
GC	gas chromatograph
GHGs	greenhouse gases
ha	hectare
HH	Human health
IRR	internal rate of return
ISO	International Organization for Standardization
ISTAT	Istituto Nazionale di Statistica/Italian National Institute of Statistics
km	kilometer
km^2^	kilometer square
kW	kilowatt
kWh	kilowatt-hour
L.L_reactor_^−1^.d^−1^	liter (of biogas or methane) per liter of or reactor content per day
L.t^−1^.km^−1^	liter per ton per kilometer
L	liter
LCA	life cycle assessment
LCC	life cycle costing
L_CH4_.L_reactor_^−1^.d^−1^	liter of methane per liter of reactor content per day
LCI	life cycle inventory
LCIA	life cycle impact assessment
LCM	life cycle management
m^3^	cubic meter
MJ	megajoule
mL.g_VS_ ^−1^	milliliter per gram of volatile solids
mL	milliliter
N_2_	nitrogen
NL.g_VS_^–1^	normal liter per gram of volatile solids
NL.kg_SV_^−1^	normal liter per kilogram of volatile solids
NL.L^−1^	normal liter per liter
Nm^3^.kg_VS_^–1^	normal cubic meter per kilogram of volatile solids
NPV	Net Present Value
OMSW	olive mill solid waste
OMWW	olive mill wastewater
p	power
pH	potential of hydrogen
PPs	polyphenols
r	discount rate
St. Dev	standard deviation
t	ton
TC	total carbon
TCD	thermal conductivity detector
t^−1^km^−1^	ton per kilometer
TN	total nitrogen
v/v	volume per volume
VS	volatile solid
